# Generation and characterization of a IgG monoclonal antibody specific for GM3 (NeuGc) ganglioside by immunizing β3Gn-T5 knockout mice

**DOI:** 10.1038/s41598-018-20951-8

**Published:** 2018-02-07

**Authors:** Dongwei He, Xiaoyan Fan, Boyi Liu, Yiqing Tian, Xiangmei Zhang, Lin Kang, Yan Tai, Shuzhen Liu, Qian Wang, Qingxia Li, Jianhui Cai

**Affiliations:** 1 Department of Clinical Bio-Cell, 4th Hospital, Hebei Medical University, Shijiazhuang, 050000 China; 2 Department of Oncology, Hebei General Hospital, Shijiazhuang, 050000 China; 3 Department of Neurobiology and Acupuncture Research, The Third Clinical Medical College, Zhejiang Chinese Medical University, Hangzhou, 310053 China; 4 Department of Nursing, Hebei General Hospital, Shijiazhuang, 050000 China; 5 Research Center, 4th Hospital, Hebei Medical University, Shijiazhuang, 050000 China; 6 Department of pathology, Hebei General Hospital, Shijiazhuang, 050000 China; 7 Laboratory and Equipment Administration, Zhejiang Chinese Medical University, Hangzhou, 310053 China

## Abstract

A murine monoclonal antibody (MAb-1) specific for GM3 has been generated by immunizing β3Gn-T5 knockout mice with purified GM3 ganglioside. The binding specificity of MAb-1 (IgG_3_ subclass) was established by an enzyme-linked immunosorbent assay (ELISA) and FACS and the antibody showed high binding specificity with GM3. Cell viability assay showed that MAb-1 significantly suppressed cell growth. Immunohistochemistry analysis revealed that MAb-1 was strongly expressed in human ovarian cancer tissues, whereas it was hardly expressed in normal tissues. Finally, antibody-dependent cellular cytotoxicity (ADCC) activities were determined by measuring lactate dehydrogenase (LDH) releasing assay and the results showed high ADCC activities in two representative ovarian cancer cell lines (OVHM and ID8). All of these data indicate that MAb-1 may be potentially used as a therapeutic antibody against ovarian cancers in clinical trials.

## Introduction

Gangliosides, a kind of sialic acid-containing glycosphingolipids, are highly enriched in the central nervous system of vertebrates^1,2^. They are essentially located on the outlet of the cell membrane in various organs and tissues. Gangliosides are suggested to mediate a variety of cell functions, including cell-cell recognition, cell growth, cell adhesion, transmembrance signaling^3–6^, etc. Recently, growthing evidence have shown that the expression of gangliosides is increased in several pathological conditions, such as neurodegenerative disorders, immune diseases and tumors^7–11^. For example, many studies have established that gangliosides are targets of active specific immunotherapy in colon carcinoma, glioblastomas^12,13^, pancreatic adenocarcinoma and melanoma^11^. However, most of the monoclonal antibodies used in these studies showed relatively low binding affinity against gangliosides because they are of the IgM subclass^14–16^. Recently, we and other teams have established that gene-engineered mice may be useful for the generation of IgG antibodies due to their absence of some series of glycosphingolipids^17–22^.

**Figure 1:**
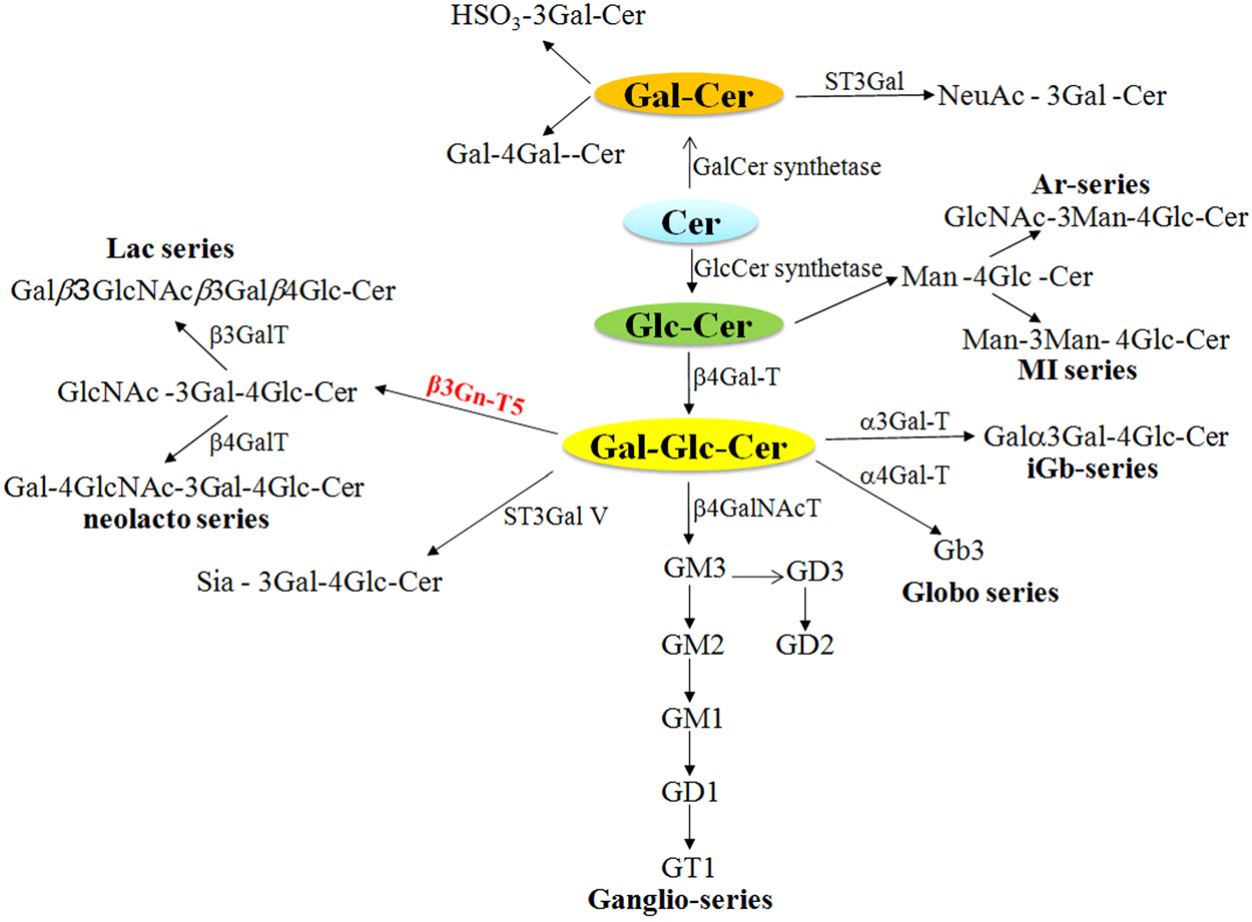
Synthetic pathway of lacto-/neolacto-series gangliosides. β3Gn-T5 synthesizes GlcNAc β1,3Galβ1,4Glc-ceramide to initiate the formation of lacto-/neolacto-series glycosphingolipids by transferring GlcNAc in a β1,3-linkage to lactosylceramide. Most glycosphingolipids were synthesized via Glc-Cer, while others were synthesized via Gal-Cer pathway. Cer, ceramide; Glc-Cer, glucosylceramide; Gal-Cer, galactosylceramide. In addition, β3Gn-T5 was shown red in the figure.

The Lc3-synthase (β1,3-N-acetylglucosaminyltransferase-V:β3Gn-T5) is the key enzyme that controls the expression of lacto-/neolacto-series glycolipids by transferring GlcNAc in a β1,3-linkage to lactosylceramide (Fig. 1)^23^. The β3Gn-T5 is highly expressed during mouse development. It becomes mostly active on embryonic day 15, then decreases to a low level, and finally locates mainly in the spleen and placenta of adult mice^23^. Recently, we have established that β3Gn-T5 knockout mice showed about 2–8 times higher response when immunized with anti-glycolipid antigens compared with C57BL/6-background wild-type mice^22^. A similar result was obtained in another independent study^16^. All of these studies suggest that β3Gn-T5 mice may be suitable animals for the generation of ganglisosides specific-monoclonal antibodies.

In the present study, we generated an anti-GM3 ganglioside monoclonal antibody (MAb-1) by immunizing β3Gn-T5 knockout mice with purified GM3 ganglioside. Furthermore, we determined the antibody specificity and reactivity. Our data indicated that MAb-1 may be a potential therapeutic antibody against human ovarian cancers in clinical trials.

## Materials and Methods

### Ethics statement

The experimental protocol was approved by Hebei General Hospital. The experimental methods and protocols were carried out in accordance with the approved guidelines and regulations. The animals used in this study were conducted under the guidelines for Animal Welfare and Experimentation of Hebei General Hospital.

### Animals

β3Gn-T5 knockout mice were bred and maintained under special pathogen-free (SPF) conditions as described before^22^. C57BL/6J mice were purchased from Experimental Animal Center, Hebei Medical University. They were bred and maintained under the same conditions of β3Gn-T5 knockout mice.

### Cell lines

Mouse ovarian cancer cell line OVHM was a gift from Dr. Hiromi Fujiwara (Osaka University, Osaka, Japan) and were cultured in RPMI1640 medium (Sigma-Aldrich, St. Louis, MO, USA) containing 10% fetal calf serum (Sijiqing Biological Engineering Materials CO., Ltd, Zhejiang, China) in our lab. Mouse ovarian cancer cell line ID8 was contributed by Professor Jianxin Cheng (Department of Gynecology, 4th hospital, Hebei Medical University, Shijiazhuang, China). ID8 cells were maintained in Dulbecco’s Modified Eagle’s Medium (DMEM, Sigma-Aldrich) supplemented with 4% fetal bovine serum, 100 U/ml penicillin, 100 µg/ml streptomycin, 5 µg/ml insulin, 5 µg/ml transferring and 5 ng/ml sodium selenite. Chinese hamster ovary (CHO) cells (Cell Bank of Type Culture Collection of Chinese Academy of Sciences, Shanghai, China) were cultured in RPMI1640 containing 10% fetal calf serum. The human epidermoid carcinoma cell line, A431 cell line (Cell Bank of Type Culture Collection of Chinese Academy of Sciences, Shanghai, China) was cultured in DMEM supplemented with 10% fetal bovine serum. All of the cells were maintained in a humidified atmosphere of 5% CO_2_ at 37 °C.

### Drug preparations

Purified GM3 (NeuGc) ganglioside was isolated from horse erythrocytes as described previously^24^. GM3 (NeuAc), GM2, GM1, GD2, GD1b, GT1b were also purchased from Sigma-Aldrich (St.Louis, MO, USA). GD3 and Gb3 were gifts from Professor Zhongning Zhu (Basic Medical College, Hebei Medical University). GM2 (NeuGc) was obtained from liver of Balb/c mice as described before^25^. Sino Biological Mouse Mab Antibody isotyping kit was purchased from Santa Cruz Biotechnology, Inc. (Dallas, TX, USA).

### Immunization of mice

8-week-old mice were immunized from the tail vein with liposomes (Sigma-Aldrich) containing GM3 ganglioside (100 μg) on day 1, 4, 8, 12, 16 and 20. The antibody titer in mouse serum was determined using ELISA assay. The details have been previously described^17–22^.

### Enzyme-linked immunosorbent assay (ELISA)

Gangliosides (2 ng/μl) were plated in 96-well plates. After drying up in the air, 5% BSA was added for 2 h at room temperature. A series of diluted MAb-1 were added to the plates and incubated for another 2 h, followed by horseradish peroxidase (HRP)-anti-mouse IgG (Amersham Biosciences) as a secondary antibody. Finally, 10 μl of substrate solution [ortho-phenylene diamine (2 mg) (Sigma) and H_2_O_2_ (8 μl) (Sigma) in 5 ml of citrate-phosphate buffer] was added to stop the reaction. The optical density was recorded at 450 nm with a scanner. The isotype control of mouse IgG_3_ was purchased from Santa Cruz Biotechnology, Inc. (Dallas, TX, USA).

### Flow cytometry

Cell surface expression of GM3 was determined by FACSCaliver^TM^ (Becton Dickinson). Briefly, about 1 × 10^6^ cells were incubated with MAb-1(10 μg/ml) or isotype control(10 μg/ml) for 60 min on ice and then stained with FITC-conjugated goat anti-mouse antibody (H + L) (Cappel, Durham, NC) for 45 min. Finally, the CELLQuest^TM^ program (Becton Dickinson, New Jersey, USA) was used to determine the positive cells.

### Measurements of affinity of MAb-1

The affinity of MAb-1 antibody against GM3 ganglioside was determined using BIAcore 3000 system (BIA core, Piscataway, NJ). Firstly, the MAb-1 antibody was immobilized on the surface of biosensor chips and coupled with N-ethyl-N′-(3-dimethylaminopropyl) carbodiimide/N-hydroxysuccinimide according to the instructions of the manufacturer. Then, 1% BSA was used as a control. The affinity rate constants (association rate constant, K_a_; disassociation rate constant, K_d_) were determined. Finally, the affinity of MAb-1 (K) were calculated as K = K_a_/K_d_.

### Cell viability assays

The cell viabilities of MAb-1 on OVHM and ID8 cells were determined by MTS (3-(4, 5-dimethylthiazol-2-yl)-5-(3–carboxymethoxyphenyl)-2-(4- sulphophenyl)-2H-tetazolium) assay according to the manufacturer’s instructions (Promega, USA). Cells were seeded in triplicate in 24-well plates and incubated overnight. Then cells were treated with MAb-1 at doses of 10, 20 and 40 μg/ml, respectively. After 0, 24, 48 and 72 h incubation, MTS solution was added to each well and incubated for 3 h at 37 °C in a humidified incubator. Finally, the absorbance at 490 nm was measured.

### Immunohistochemistry analysis by MAb-1

Immunohistochemistry analysis was performed as follows. Briefly, 20% sucrose-fixed human ovarian cancer tissues were cut into 10 μm sections. Then, they were incubated with MAb-1 (15 μg/ml) or isotype control (15 μg/ml) at 4 °C overnight. Next, they were incubated with biotin-conjugated secondary antibody for 30 min at room temperature and then incubated with streptavidin-horseradish peroxidase complex for 30 min. Finally, the sections were incubated with 3, 3′-diaminobenzidine for 10 min and counterstained with hematoxylin. The immunostained slides were evaluated by two independent observers under the microscope. Positive staining was detected as a brown color of the cells. Five high-power fields were randomly selected, and the percentage of positive cells in these fields was counted. Tumors without staining or with weak staining (positive cell rate <10%) were classified as negative while tumors with moderate (10%≤ positive cell rate <75%) to intense staining (positive cell rate ≥75%) were classified as positive.

### Isolation of peripheral blood mononuclear cells (PBMCs)

PBMCs of mice were isolated as follows: Firstly, 5 ml peripheral blood was collected and diluted by addition of an equal volume of 1× PBS. Then the diluted blood was carefully loaded over an equal volume of lymphoprep reagent (Mouse Lymphoprep Reagent kit, Beijing Solarbio Science & Technology Co., Ltd) in a 50 ml centrifuge tube. After centrifugation at 800 g for 20 min at room temperature, the distant band at the blood/lymphoprep was carefully transferred into a new tube. The harvested fraction was washed twice and finally a pellet of cells was obtained.

### ADCC assay

OVHM and ID8 cells were used as target cells and fresh PBMCs were used as effector cells. The ADCC was evaluated using a LDH release assay (Promega, Madison, USA) in 96-well plates, according to the manufacturer’s protocol. Briefly, cells were incubated at 37 °C for 20 h. Then, 100 ng/ml MAb-1 was added into each well. Next, the effector cells were added into the well with the E:T ratios of 200:1, 100:1, 50:1, 20:1, 10:1, 5:1 and 2:1, respectively. After 4 h co-incubation, LDH release in the supernatants was determined at 490 nm. The percentage of cellular cytotoxicity was calculated using CytoTox 96 Non-Radioactive Cytotoxicity Assay^TM^ (Promega) according to the manufacturer’s instructions. Data were graphed and analyzed using GraphPad Prism5.0.

### Statistical analysis

The results were reported as mean ± SD. Statistical significances were performed by one-way analysis of variance (ANOVA) followed by Tukey post hoc test between groups and multiple comparisons. A p value of less than 0.05 was evaluated as statistically significant.

## Results

### Generation of an anti-GM3-specific monoclonal antibody

**Figure 2:**
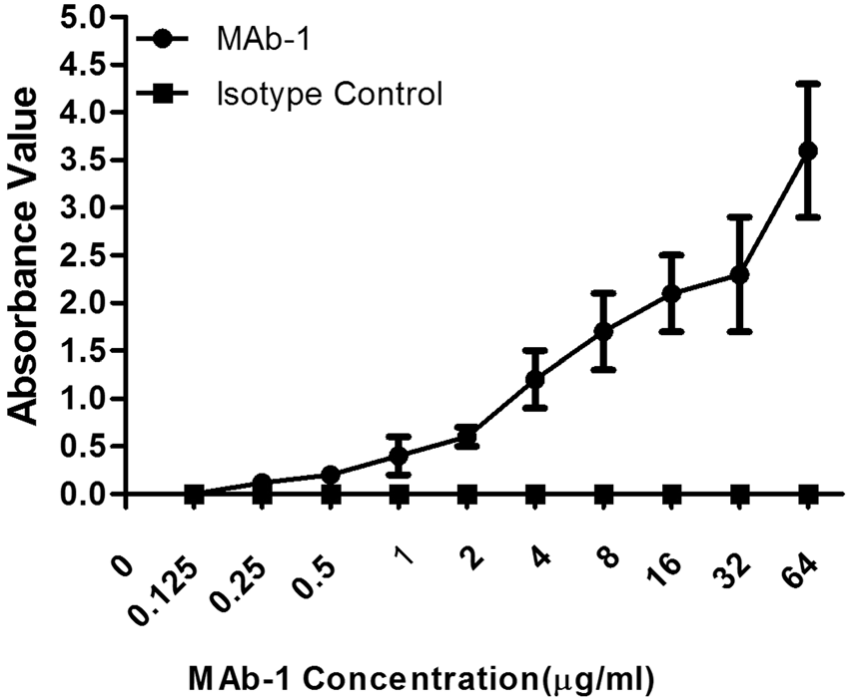
MAb-1 reacts with GM3 in a dose-dependent manner in ELISA. GM3 ganglioside was immobilized in the plates and incubated with diluted MAb-1 concentrations. Mouse IgG_3_ purchased from Santa Cruz Biotechnology was used as isotype control. The data presented are means ± SD from at least three independent experiments.

After immunization of 3 mice with GM3 ganglioside embedded in liposome, the titers in each mouse were determined by ELISA and the mouse with the highest titer of anti-GM3 were chose for the following experiment. Spleen cells were fused with NS-1 myeloma cells. After hypoxanthine-aminopterin-thymidine (HAT) selection, a number of Mabs reactive with GM3 ganglioside were generated. Briefly, about 316 clones were available from 921 clones, and 89 definitely positive clones were identified by immuno-fluorescence (IF) assay. Furthermore, these clones were subcloned and checked by IF assay again. Finally, only 13 clones were found to be significantly positive with glycolipids (data unpublished). Among them, MAb-1 was firstly established and tested on tumor immunity against ovarian carcinoma. As shown in Fig. 2, MAb-1 reacted with GM3 in a dose-dependent manner in ELISA.

### Specificity of MAb-1 against GM3 at cellular level

**Figure 3:**
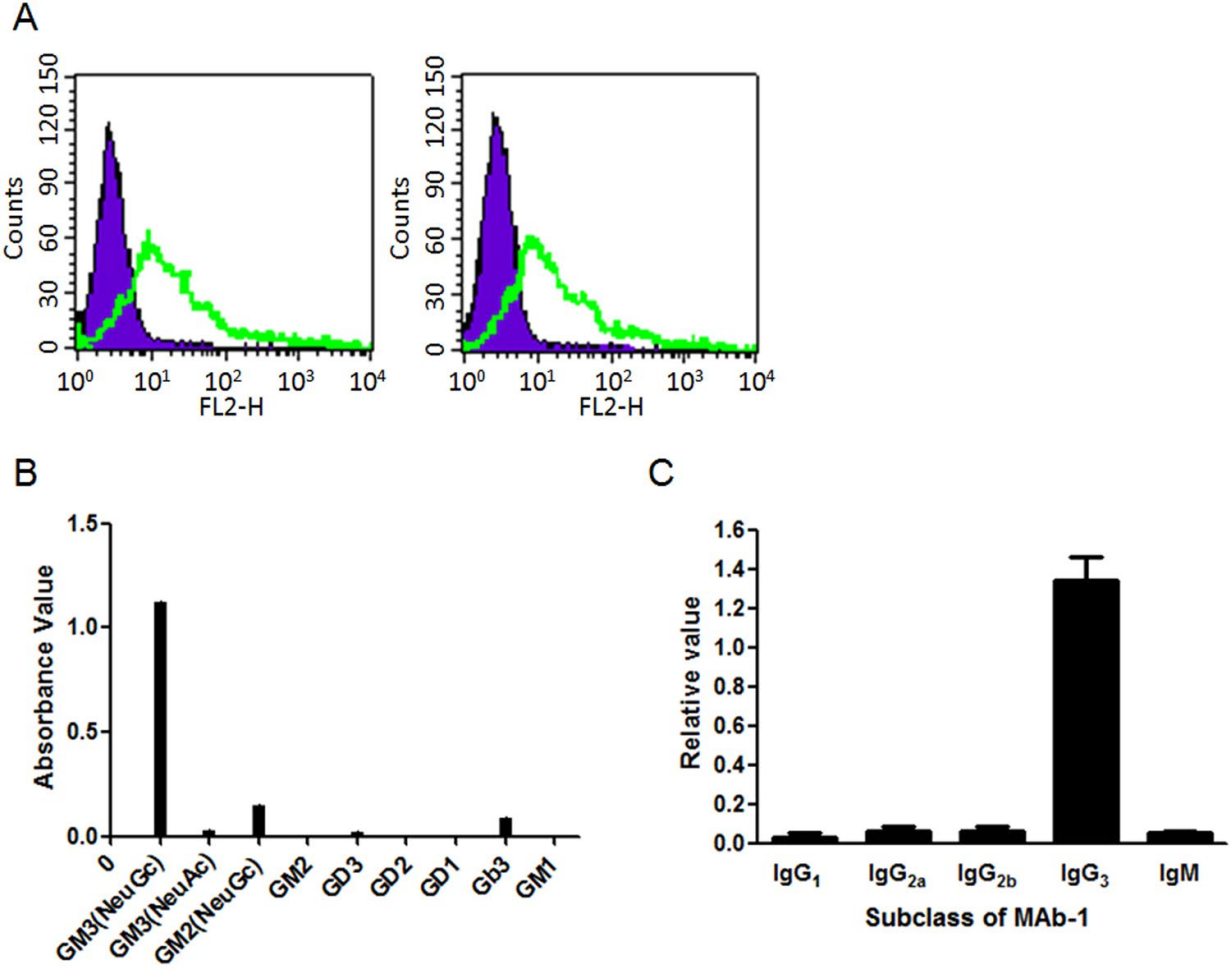
Characters of Mab-1 antibody. (**A**) Specificity of MAb-1 against CHO cells (left panel) and A431 cells (right panel). The purple (left) and the green (right) lines in each panel represent the control and Mab-1 groups, respectively. (**B**) Epitope determination of Mab-1 by ELISA assay. Nine gangliosides were immobilized and followed incubation with purified MAb-1 antibody. (**C**) Subclass of MAb-1. The method was performed according to the guide of Sino Biological Mouse Mab Antibody isotyping kit.

Flow cytometry was used to determine the specificity of MAb-1 at cellular level (Fig. 3A). The purple portion indicates the negative control and the green portion indicates the positive expression of GM3 ganglioside. Both CHO and A431 cell lines, which endogenously express GM3 gangliosides, were recognized by MAb-1, indicating identical specificity of MAb-1 against GM3 at cellular level.

To further analyze the epitope of MAb-1 antibody, the antibody was tested against other a-series (GM2, GM1), b-series gangliosides (GD3, GD2, GD1), globo-series(Gb3) as well as against GM3 (NeuAc). As shown in Fig. 3B, no cross-reaction with other gangliosides was found. Moreover, the association and dissociation rate constants (K_a_ = 6.08 × 10^4^ (mol/l s)^−1^ and K_d_ = 3.17 × 10^−4^ s^−1^) were determined, respectively. The affinity of MAb-1 (K = K_a_/K_d_) was calculated as 1.92 × 10^8^ (mol/l)^−1^. Similarly, the affinity of 14F7 against GM3 ganglioside was 1.79 × 10^8^(mol/l)^−1^, indicating that it has similar affinity compared with MAb-1.

### Subclass of MAb-1 antibody

Next, the subclass of MAb-1 antibody was established using Sino Biological Mouse Mab Antibody isotyping kit (Fig. 3C). The results clearly showed that the subclass of MAb-1 was IgG_3_.

### Cell viabilities inhibitions induced by MAb-1

**Figure 4:**
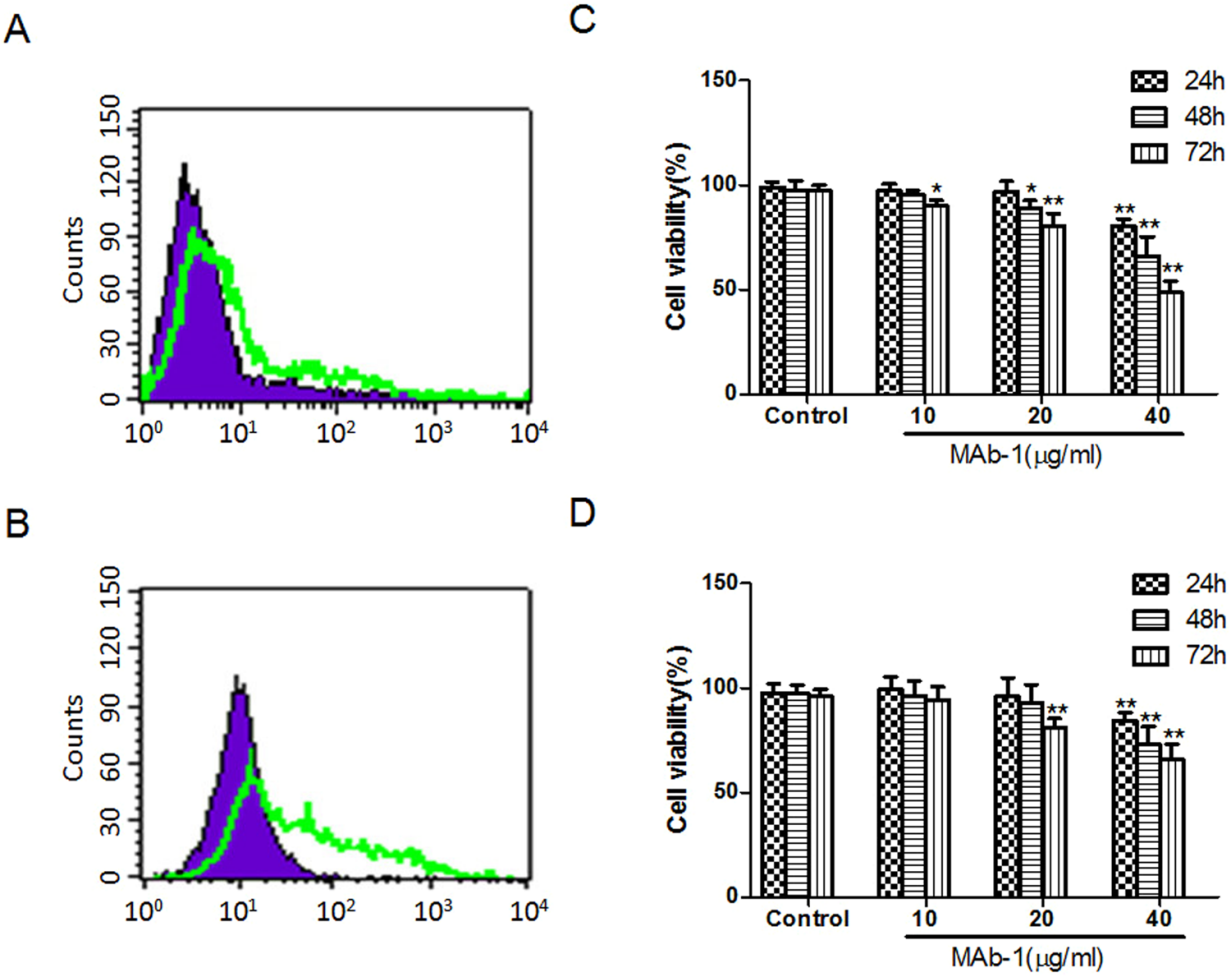
MAb-1 inhibited cell viabilities of OVHM and ID8. Expression of GM3 ganglioside in mouse ovarian OVHM (**A**) and ID8 (**B**) cells. Cell viabilities were inhibited by MAb-1 in OVHM (**C**) and ID8 (**D**) cells. All data represent mean ± SD from at least three independent experiments. *p < 0.05, **p < 0.01 compared with control groups, respectively.

To investigate the role of MAb-1 in cell viabilities, 2 mouse ovarian cancer cell lines (OVHM and ID8) were firstly treated with MAb-1 to check the expression of GM3 ganglioside and the results were shown in Fig. 4A,B. 10, 20 and 40 μg/ml of MAb-1 were used to determine cell viabilities (Fig. 4C,D). Compared with control cells, 10 μg/ml MAb-1 barely showed any effect on OVHM cells at 24 h and 48 h, while it only showed a little effect at 72 h in OVHM cells (p < 0.01). At 20 and 40 μg/ml, MAb-1 significantly inhibited cell proliferation rates at 48 h and 72 h (p < 0.01). Furthermore, the inhibitory effects showed time- and dose-dependent manners.

### Immunohistochemistry analysis by MAb-1 against ovarian tissues

**Figure 5:**
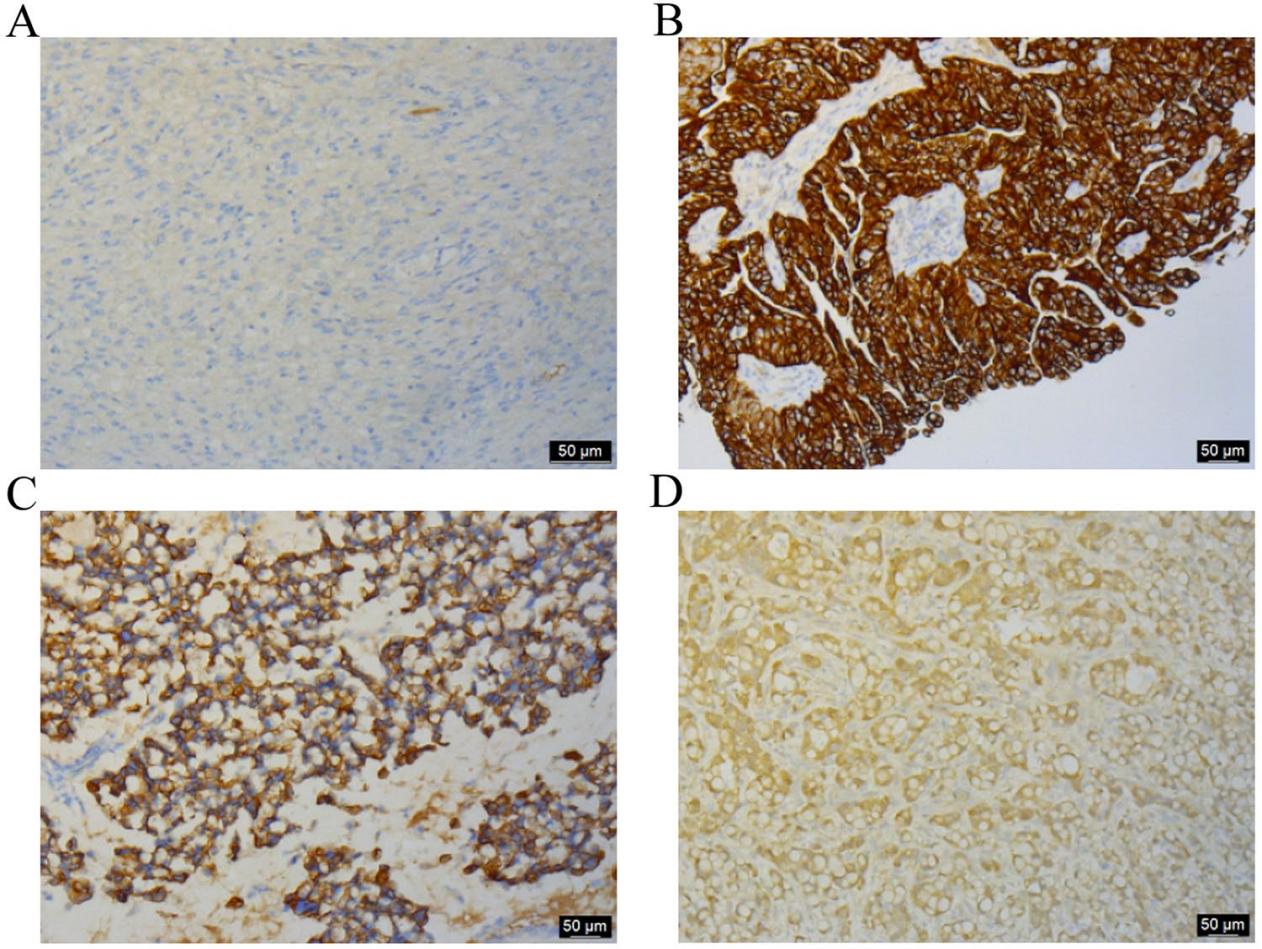
Immunohistochemistry analysis by MAb-1 against human ovarian tissues. Representative images are shown (100×). (**A**) Normal ovary, (**B**) serous adenocarcinoma, (**C**) mucinous adenocarcinoma, (**D**) metastatic adenocarcinoma.

Since GM3 ganglioside is known to be strongly expressed in human ovarian cancer cells, immunohistochemistry analysis was performed to detect the immunoreactivity of MAb-1 on human ovarian cancer tissues. In total, 56 human ovarian cancer samples were checked and 43 were positive. This result indicated the immunoreactivity was about 76.79%. Immunostaining by MAb-1 was negative in normal ovarian tissues (Fig. 5A), but showed strong staining in a cell-surface and cytoplasm pattern in ovarian cancer cells, such as serous adenocarcinoma (Fig. 5B), mucinous adenocarcinoma (Fig. 5C) and metastatic adenocarcinoma (Fig. 5D). These results suggested that MAb-1 specifically reacted with GM3 ganglioside in human ovarian cancer tissues.

### ADCC by MAb-1

**Figure 6:**
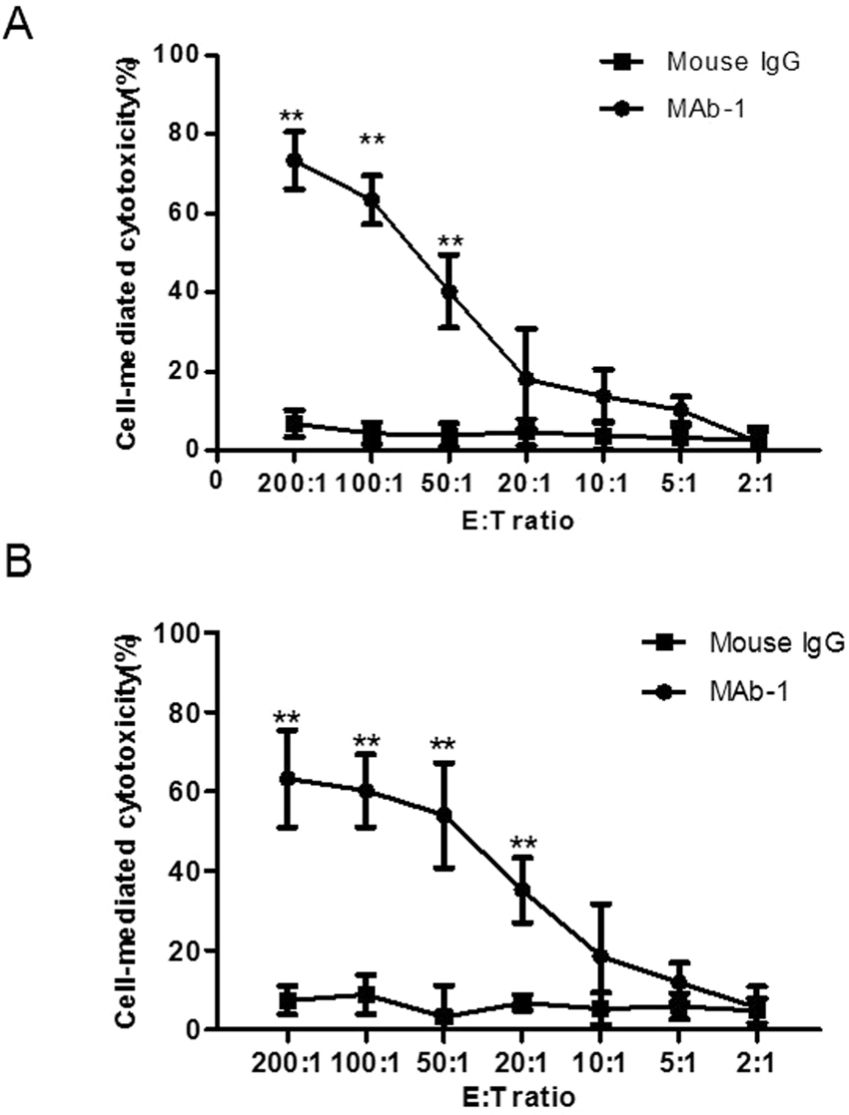
ADCC mediated by MAb-1 or control IgG in mouse ovarian cancer cell lines OVHM and ID8 assessed using LDH release assay. E:T ratio was 200:1, 100:1, 50:1, 20:1, 10:1, 5:1 and 2:1. (**A**) OVHM cell line. (**B**) ID8 cell line. All data represent mean ± SD, **p < 0.01 compared with control groups. Results are representative of three independent experiments.

ADCC effects induced by MAb-1 were evaluated by determining the activity of cytosolic LDH released by treating OVHM and ID8 cells, respectively. As shown in Fig. 6, (1) the results of cell-mediated cytotoxicity (%) in these two cell lines all reached maximum at E:T ratio of 200:1; (2) At E:T ratio of 200:1, 100:1 and 50:1, the cytotoxicity (%) in OVHM and ID8 cells showed significantly higher levels compared with that of control groups (p < 0.01); (3) The cytotoxicity showed an E:T ratio-dependent manner when treated with 100 ng/ml MAb-1; (4) The control mouse IgG did not cause any significant cell lysis (less than 10%). These results indicated that MAb-1 could generate specific ADCC effects in mouse ovarian cancer cells.

## Discussion

Since 1985, it has been well known that glycosphingolipids are ubiquitous membrane components of various organs^2^. Although they are natural components of the plasma membranes of vertebrates, numerous evidence indicate that gangliosides are attractive targets for immunotherapy due to different expression patterns during oncogenesis and tumor development^26–30^. Some of these gangliosides have been used as tumor markers or tumor-associated antigens in cancer diagnosis and therapy^31–33^. Especially, GM3 (NeuGc) is one of the most common types of sialic acid, which scarcely exists in normal human tissue but strongly expresses in glycoconjugates of human tumors^34,35^. These studies indicate that GM3 can be a potentially attractive target for cancer diagnosis and therapy. In view of the above points, a large number of antibodies, such as L612^36^, GMR6^15^, 8G9D8^37^, AbFCM1^38^, have been established against NeuGc-containing gangliosides. At present, it is a little difficult for us to obtain these antibodies. However, we found that the subclass of L612, 8G9D8, GMR6 and AbFCMl was IgM, but not IgG subclass. In the application of reacting cancer cells with mAbs, IgG subclass mAbs are preferable, since they can be easily purified and possess immunological actions such as ADCC. Also, GMR6 exhibited broader specificities with GM4, GM1b, GD1a and GT1b. In addition to ganglioside GM3, 8G9D8 may bind to glycoproteins or another glycolipid of the stratum corneum in normal skin with a shared carbohydrate sequence. All of the above results have shown that MAb-1 monoclonal antibody in this study has valuable advantage in the future. Recently, 14F7, a IgG1 binding NeuGc-GM3 monoclonal antibody, was proved to bind specifically to GM3 (NeuGc) in breast, melanoma, colon and primary lymphoid tumors^39–41^. These results suggest that novel IgG antibodies specific to GM3 ganglioside can be reasonable and practicable.

In the present study, we used purified GM3 ganglioside to immunize β3Gn-T5 knockout mice to generate IgG monoclonal antibodies (IgG_3_ subclass) against human ovarian cancer tissues. β3Gn-T5 knockout mice, which lack Lc3-synthase, the key enzyme that controls the expression of lacto-/neolacto-series glycolipids, at some point, can enhance the antigen specificity. ELISA assay has shown that MAb-1 reacted with GM3 in a good dose-dependent manner. This result was also obtained by another independent study^16^. Furthermore, at cellular levels, the newly generated MAb-1 can significantly recognize cell lines that highly expressed GM3. These results further suggest that β3Gn-T5 knockout mice can be suitable animals for generating anti-glycolipid antigens with lacto-/neolacto-series structures.

It is generally accepted that NeuAc-gangliosides are expressed in normal tissues. Most studies have shown that NeuGc-gangliosides are widely expressed in human tumors^39,42,43^ and cell lines^44^. To our best knowledge, this is the first description of a murine IgG3 mAb specific for GM3 by immunizing β3Gn-T5 knockout mice. MAb-1 reacted with GM3 (NeuGc), but not GM3(NeuAc), suggesting that NeuGc structure affected MAb-1 binding. On the other hand, MAb-1 showed a little reactivity with GM2 ganglioside, indicating that the GalNAcβ1–4 residue significantly affect the binding efficacy of Mb-1. As for 14F7 antibody, similar results were found before^27^. Both of them showed specific reactivity with GM3 (NeuGc) ganglioside, suggesting that GM3(NeuGc) could be a suitable epitope.

It has been reported that most gangliosides (such as GM1, GD1a and GD3) of tumors, especially tumor microenvironment, can positively influence tumor growth^10^. As for GM3 ganglioside, early studies showed that exogenous GM3 inhibited the cell proliferation of several cancer cells^43,45^. However, the growth of the Siat9 (encoding GM3 synthase) and Galgt 1(encoding GM2 synthase)-deficient knockout tumor cells is significantly impeded both *in vivo* and *in vitro*^10,46^. The current data seem to suggest that the role of GM3 ganglioside in cell proliferation remains controversial. Here, in 2 ovarian cancer cell lines, MAb-1 treatment significantly inhibited the cell proliferation and showed good dose- and time- dependent manners. The discrepancies are likely due to the different concentrations of GM3 being used, different cell lines and tumor types and/or different administration of ganglioside-specific IgG antibodies.

It has been well established that ADCC is one of the immune effector mechanisms associated with antibodies against tumor-associated gangliosides^47,48^. The IgG Fc domain can interact with human FcγRs on effector cells and IgG_3_ is considered one of the principal human isotypes for activating FcγRs^49^. In the present study, the subclass of MAb-1 is IgG_3_ subclass and we further assessed the ability of MAb-1 to induce ADCC in ovarian cancer cells. Our results indicate that MAb-1 can induce remarkable ADCC effects on effector cells.

In conclusion, by immunizing β3Gn-T5 knockout mice with purified GM3 ganglioside, we successfully generated MAb-1 antibody. The MAb-1 reacted with GM3 in a dose-dependent manner in the ELISA assay. Furthermore, we detected the specific reaction of the antibody against ovarian cancer cells via immunohistochemistry and ADCC analysis. These results indicate that MAb-1 is a potentially effective IgG monoclonal antibody which may further be used in antibody-dependent diagnose and therapy of ovarian cancers.
